# Factors influencing smart device addiction among preschool children: An extended protection-risk model perspective

**DOI:** 10.3389/fpsyg.2023.1017772

**Published:** 2023-02-09

**Authors:** Lu Cheng, Junwei Cao

**Affiliations:** ^1^Department of Child and Family Studies, Kyungpook National University, Daegu, Republic of Korea; ^2^School of Business, Yangzhou University, Yangzhou, China

**Keywords:** emotion regulation, parents, smart device addiction, preschool children, protection-risk model

## Abstract

Today, users of smart devices are from increasingly younger generations, and their use is very common among preschoolers. The problem of smart device addiction in preschool children has attracted widespread attention, due to which this study explores the influencing factors in children aged 2–5 years. Based on the protection–risk model, 236 Chinese parents were surveyed, and the data was analyzed using partial least squares structural equation modeling. The findings show that parental emotion regulation significantly and negatively influences children’s depression and social withdrawal symptoms, whereas it has a significant and positive influence on parental self-control as well as outdoor intention. Also, children’s depression and social withdrawal symptoms have a significant and positive influence on their smart device addiction, whereas parental self-control and outdoor intentions have no significant influence on it. Moreover, children’s social withdrawal and depression have a mediating effect between parental emotion regulation and children’s smart device addiction, but parental self-control and outdoor intention have no mediating effect between the two. This study identifies the factors influencing children’s smart device addiction from a new perspective, providing theoretical support to address this problem of addiction.

## 1. Introduction

Addiction is defined as being excessively devoted to something in which one loses the ability to make free choices or becomes a slave (Sharma et al., 2021). Addiction can be in terms of both drugs and behavior. The former is a neuropsychiatric disorder characterized by the repeated use of drugs despite harmful consequences, whereas the latter is similar to drug addiction and includes addiction to gambling, food, the Internet, and mobile phone (Zou et al., 2017). Among these, mobile phone addiction is of particular concern (Bianchi and Phillips, 2005), as it has become a new and increasingly prominent societal problem (Shapira et al., 2003; Zou et al., 2017). As digital technology offers convenience, people have started using smart devices more frequently, due to which mobile phone addiction has evolved into smartphone addiction (Bozzola et al., 2018; Sharma et al., 2021; Lee et al., 2022). Smartphone addiction is defined as a behavior characterized by the overuse of smartphones (Aljomaa et al., 2016).

Smart devices represented by smartphones strongly attract the attention of preschoolers (Bozzola et al., 2018). According to Park and Park (2021), one in five preschoolers who use smart devices may be addicted to them. This is because preschoolers’ cognitive characteristics are less proficient than those of adults, making them more prone to be addicted to smart devices (e.g., smartphones and tablets) (Bjorklund and Green, 1992; Yang et al., 2022). Park et al. (2018) categorized screen use in children aged 2–5 years for more than 1 h per day as screen overuse. Excessive use of smart devices by children can have a serious impact on their physical and psychological health (Park and Park, 2021). The World Health Organization (WHO) recommends that young children should be allowed screen time of less than 1 h a day because when exceeded, their health and behavior can become problematic (WHO, 2019). Preschoolers’ media use of more than 1 h a day is associated with poorer cognitive, language, and social–emotional skills (Cho and Lee, 2017; Bozzola et al., 2018; Lin et al., 2020; Schwarzer et al., 2021). Children’s social competence and emotional intelligence, opportunities to interact with peers, and physical activity decrease as they become addicted to smart devices (Cho and Lee, 2017; Domoff et al., 2019; Lin et al., 2020), ultimately interfering with their learning development (Bozzola et al., 2018). The overuse of smartphones by children between 1 and 6 years of age not only affects their sociability and activity but also increases their emotional sensitivity (Lee et al., 2022). In addition, addiction to smart devices can interfere with family harmony causing parent–child conflict (Domoff et al., 2019). One study indicated that electronic devices could interrupt conversations or activities between parents and preschoolers up to 12–16 times a day (Carson and Kuzik, 2021). Therefore, it is important to understand the factors exacerbating or reducing children’s addiction to smart devices (Yang et al., 2022).

The currently available literature explains the elements of children’s smart device addiction in terms of parental and child factors. In terms of child factors, 2-year-old toddlers with self-regulation difficulties view more television and videos (Radesky et al., 2014). Moreover, children’s externalizing behaviors can also drive their addiction to smart devices (McDaniel and Radesky, 2020). However, few studies have empirically investigated their psychological factors (e.g., depression and social withdrawal) in relation to their smart device addiction. In terms of caregiver factors, parenting stress and styles are important in children’s smart device addiction (McDaniel and Radesky, 2020; Lee et al., 2022; Lee and Kim, 2022; Yang et al., 2022). Studies have also identified a strong relationship between mothers’ negative parenting behaviors (e.g., overprotection, permissibility, rejection, and neglect) and preschoolers’ over-dependence on smart devices (Lee and Kim, 2022). In addition, parents’ education level, family income, and employment status are also important factors (Livingstone et al., 2015; Cho and Lee, 2017; Park and Park, 2021). One study has even suggested that most parents of smartphone-addicted children (1–6 years old) come from a lower educational background and lack stable employment (Cho and Lee, 2017). It has also been suggested that when parents overuse smartphones to relieve parenting stress, children are also likely to do the same (Lee et al., 2022). Preschoolers are at an early developmental stage, and their behavior often imitates that of their parents (Konok et al., 2019). Therefore, to solve the problem of children’s smart device addiction, we must focus on parental factors.

It has been suggested that parental emotion regulation skills are closely related to children’s behavior and psychology. A study of parents of children aged 2–8 years found that formers’ low emotional regulation skills were significantly associated with stress in the latter (stress, agitation, fear of separation, etc.) (Shorer and Leibovich, 2020). Another study proposed that parents’ negative emotional expressiveness was not only associated with disruptive behavioral problems in children aged 5–9 years but also had an impact on their ability to regulate their emotions (Duncombe et al., 2012). These findings suggest that improving parental emotion regulation may help address children’s smart device addiction; however, there is a lack of empirical evidence regarding the relationship between the two, and therefore, further research is warranted. Thus, this study raises the following question: What is the relationship between parents’ emotional regulation and their children’s smart device addiction?

This study builds a model based on the protection–risk model, developing hypotheses accordingly. The data was obtained from a survey carried out, with the parents of preschool children acting as respondents. The potential contributions of this study are as follows: (1) This study applies the protection–risk model to preschoolers’ smart device addiction, adding to the literature and broadening the scope of application. (2) Valuable suggestions have been provided to reduce preschool children’s addiction to smart devices. (3) This study provides empirical evidence for research in the field of developmental psychology, which can help deepen the understanding of preschoolers’ smart device addiction and contributes to the future development of intervention methods.

The rest of this paper is organized as follows: Section 2 presents the theoretical background, the development of the research model and hypotheses of the study is mentioned in Section 3, Section 4 describes the data collection and analysis methods, Section 5 analyzes the results which are discussed in Section 6 along with the measures to reduce addiction to smart devices in preschool children. It also includes the limitations of the study.

## 2. Theoretical background

**Figure 1:**
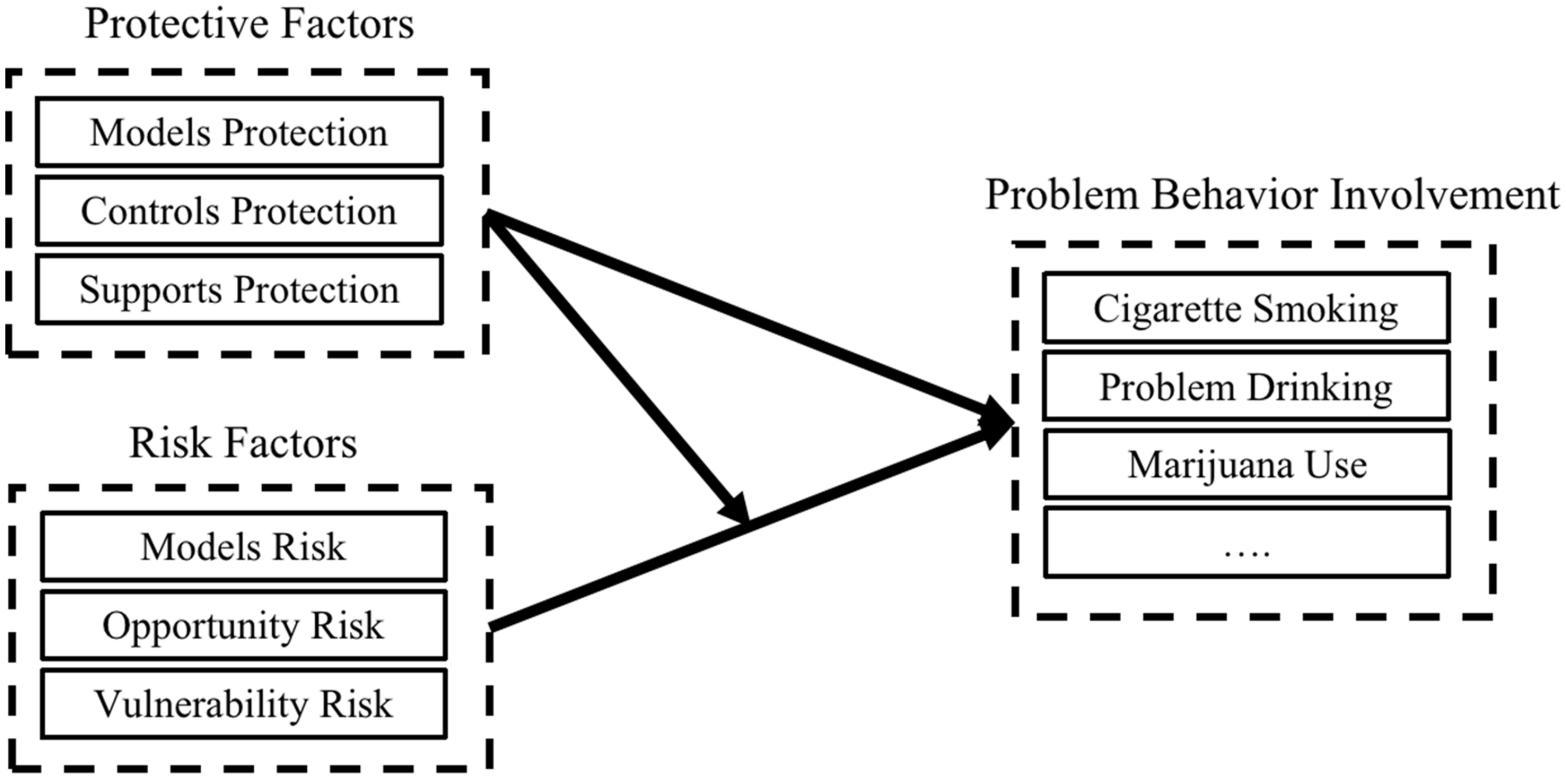
The original protection–risk model.

The protection–risk model was proposed by Jessor et al. (2003) to explain adolescent problem behavior involvement (Figure 1) and is composed of three protective and risk factors. Protective factors are those that reduce the likelihood of problem behavior by providing positive influences, including model, control, and support protection. Model protection includes parental and peer role model measures, such as healthy behaviors of parents and peers, and parental outdoor intention (motivation to implement outdoor activities) (Rhodes and de Bruijn, 2013) and self-control; control protection includes individual as well as social and environmental control measures, such as family control; and support protection includes situational support measures, such as family support (Jessor et al., 2003). Risk factors include model, opportunity, and vulnerability risks. Model risk includes measures of social role modeling, such as peer smoking; opportunity risk includes measures of opportunity, such as the presence of cigarettes at home; and vulnerability risk includes measures of tension in the family and at school (personal vulnerability) leading to depression and social withdrawal (Jessor et al., 2003). Problem behavior involvement includes involvement in problem behavior, such as crime, smoking, and alcohol (Jessor et al., 2003).

Protective factors can negatively influence the involvement of problematic behavior, while risk factors can positively influence it. When the level of protection is low, the risk factor is greater, and involvement in problem behaviors is greater; conversely, the opposite is seen when the level of protection is high. The protection–risk model explains the moderating effect of protective factors on risk factors in addition to the direct effect of protective factors and risk factors on problem behavior. The model proposes that protective factors can indirectly influence problem behavior by moderating the effect of risk factors, thus reducing their influence (Jessor et al., 2003).

This model has been applied in studies to explain students’ problem behavior involvement. A study using the protection–risk model to explain smoking behavior among college students confirmed that protective and risk factors had a significant influence on smoking among college students and that the former moderated the latter’s effects (Costa et al., 2007). The model can also explain social contexts and adolescent problem behavior (Costa et al., 2005). A study based on the protection–risk model indicated that both protective and risk factors could influence adolescents’ problem behaviors as well as pro-social behaviors (Jessor and Turbin, 2014).

However, studies on the protection-risk model have focused on adults or adolescents. To date, this model has not been applied to preschool children. This study aims to apply the protection-risk model to identify the factors influencing smart device addiction in preschool children. This study hypothesizes that model protection and vulnerability risk include parental outdoor intention and self-control as well as depression and social withdrawal in children, respectively. In addition, we hypothesize that 1. Parental outdoor intention and self-control negatively influence children’s smart device addiction, whereas children’s depression and social withdrawal positively influence the same. 2. Parental outdoor intention and self-control, as well as child depression and social withdrawal, have mediating effects in the relationship between parental emotion regulation and children’s smart device addiction.

## 3. Research model and hypotheses development

### 3.1. Research model

**Figure 2:**
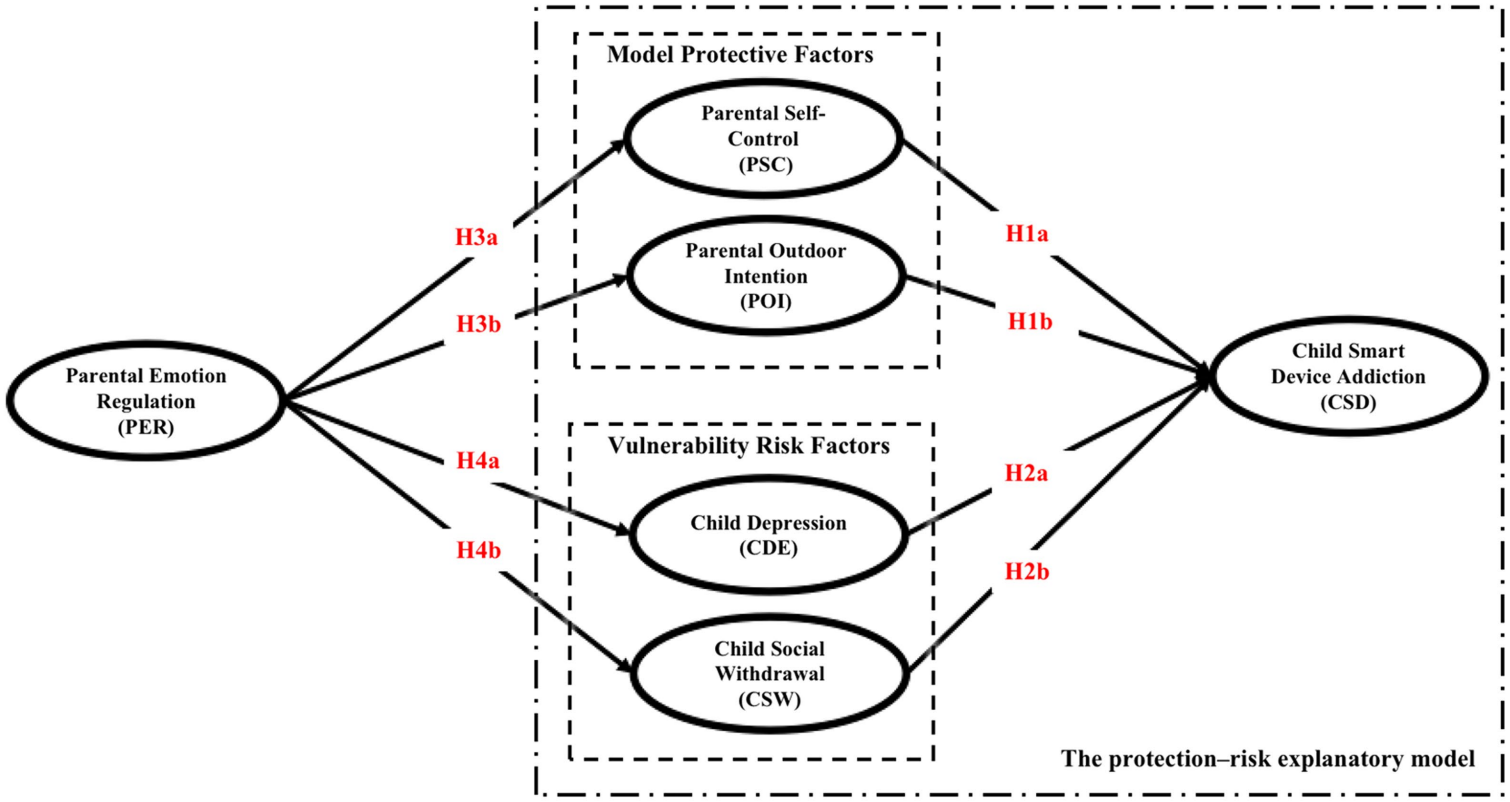
Proposed research model.

This study is based on the protection risk explanation model. The research model proposed in this study is illustrated in Figure 2. This study hypothesizes that parental emotion regulation does not directly influence preschoolers’ smart device addiction but influences parental self-control and outdoor intention of model protection, as well as preschoolers’ depression and social withdrawal vulnerability risk. These variables are associated with smart device addiction.

### 3.2. Hypotheses development

Parents with low self-control may not engage in effective parenting practices, lack awareness of their child’s deviant behavior, fail to strengthen supervision and discipline efforts, and as a result, their children are more likely to be exposed to a hostile and less nurturing family environment (Meldrum et al., 2016). Social learning theory suggests that children’s behavior can be acquired through observational learning processes and that role-model behavior influences it (Bandura, 1976). Children tend to imitate their parent’s behavior, and parents with high self-control are more likely to have children displaying the same (Nofziger, 2008; Boutwell and Beaver, 2010). Several studies have shown that parental screen time is positively correlated with that of children, and overuse of smartphones by parents may lead to similar behavior in children (Lene’McFarland, 2010; Adebar, 2018; Lee et al., 2022; Rai et al., 2022). Conversely, children tend to use their smartphones less if their parents have self-control over their own use (Cho and Lee, 2017). Therefore, the following hypothesis is proposed:

*H1a*: Parental self-control has a negative influence on smart device addiction in preschoolers.

Humans are social beings and need to belong and interact with others. When taking part in outdoor activities, people are more likely to engage face-to-face, thus, reducing the use of connected devices and social media, thereby lowering the risk of Internet addiction (Estévez et al., 2017; Helms et al., 2019; Diotaiuti et al., 2022). This is also true in the case of families, where the more time children spend outdoors, the less time they spend using screen devices (Patten et al., 2017; Hasanen et al., 2021; Nielsen and Arvidsen, 2021). Children’s behavior is shaped by family practices (Plowman et al., 2010; Adebar, 2018). If parents as role models for children demonstrate a greater willingness to engage in outdoor activities, they guide them to become more involved in such activities (Pergams and Zaradic, 2008; Schneider, 2016). Therefore, when parents have a strong intention to engage in outdoor activities, there are more of them, and in such cases, children’s addiction to smart devices may be alleviated. Therefore, we propose the following hypothesis:

*H1b*: Parental outdoor intentions have a negative influence on smart device addiction in preschoolers.

Deficiencies in personal characteristics (e.g., low self-esteem, introversion, anxiety, depression, impulsiveness) and social relationships (e.g., shyness, social phobia, loneliness, social isolation, rumination) are risk factors that can lead to Internet addiction (Estévez et al., 2017; Diotaiuti et al., 2022). Phones are a way of coping with depression and negative emotions as they can provide psychological and social support to people (Serra et al., 2021), thereby attracting use. The overuse of digital devices offers the possibility of enjoyment and escape from reality, and people may tend to overuse cell phones to compensate for lack of emotional relationships (Lee and Ogbolu, 2018).

Anxiety and depression are positively associated with addictive technology use (Andreassen et al., 2016; Houghton et al., 2018). Adolescents with high levels of depression escape negative emotions by overusing their smartphones (Mun and Lee, 2021). Children and adolescents with reduced interpersonal skills tend to spend more time on screen media devices (Lee and Ogbolu, 2018). Additionally, these devices can be used as “electronic babysitters” to distract, soothe, and accompany children (Radesky et al., 2016; Reid Chassiakos et al., 2016; Lin et al., 2020).

Smartphones can provide psychological support to children (Serra et al., 2021), as they can be used as cathartic outlets for children suffering from depression, social withdrawal, and other psychological problems arising in preschoolers. This increases the use of and psychological dependence on smart devices, leading to addiction (Shapira et al., 2003; Estévez et al., 2017). Therefore, we propose the following hypotheses:

*H2a*: Preschoolers' depression has a positive influence on their smart device addiction.

*H2b*: Preschoolers' social withdrawal has a positive influence on their smart device addiction.

Emotion regulation consists of internal (e.g., physiological reactivity and cognitive effort) and external responses (e.g., emotional expressions, facial reactions, and emotion-driven behaviors), which jointly influence the management of emotional intensity, duration, and display (Morelen et al., 2016). Emotional regulation and self-control are interrelated in everyday life, both being controlled responses rather than automatic ones (Paschke et al., 2016; Wenzel et al., 2020). Emotions can cause self-control problems (Tice and Bratslavsky, 2000; Chester et al., 2016), and when people experience negative emotions, their self-control decreases (Tice and Bratslavsky, 2000; Chester et al., 2016). Negative emotions have been known to excessively tax inhibitory areas of the prefrontal cortex, leading to a failure of self-control (Chester et al., 2016). The lower the level of emotion regulation, the lower the level of self-control. Therefore, we hypothesize:

*H3a*: Emotional regulation has a positive influence on self-control.

Research has shown a strong relationship between mental health and outdoor activities (Hanna et al., 2019). Active participation in outdoor activities has a wide range of beneficial effects on both adults and children (Lene’McFarland, 2010). Outdoor activities alleviate negative emotions and increase positive ones, which are important for emotional health and regulation (Bowler et al., 2010; Johnsen and Rydstedt, 2013; Pasanen et al., 2014). Positive emotions demonstrate an open mind and are associated with intrinsic outdoor motivation (Løvoll et al., 2017), which means that parents with higher emotional regulation skills have strong intentions to participate in outdoor activities. Therefore, this study proposes the following hypothesis:

*H3b*: Emotional regulation positively influences outdoor intention.

Parental emotion regulation is important for young children’s development as they imitate their parents’ emotional expressions (Are and Shaffer, 2015). Mothers with highly adaptive emotion regulation skills provide a positive emotional environment (Are and Shaffer, 2015). In contrast, when they are angry, they may react negatively to their children (Dix et al., 1990). Poor maternal emotion regulation may weaken the child’s ability to tolerate distress, increase their emotional arousal (Scaramella and Leve, 2004; Mirabile et al., 2009), and affect their ability to produce positive emotional responses (Crespo et al., 2017). In addition, parental emotion dysregulation is significantly associated with withdrawal and depression in children (Han and Shaffer, 2012). Lack of emotional awareness and impulse control difficulties among mothers are strongly associated with depression in children and adolescents (Gouveia et al., 2018). Thus, the level of parental emotion regulation is strongly associated with depression and social withdrawal in children. Emotionally regulated parents, who positively influence their children, reduce the likelihood of depression, social avoidance, and other problems. Therefore, we propose the following hypotheses:

*H4a*: Parental emotion regulation has a negative influence on preschoolers' depression.

*H4b*: Parental emotion regulation has a negative influence on preschoolers' social withdrawal.

## 4. Empirical analysis

### 4.1. Questionnaire survey design

To accommodate the current study, the questionnaire variables were adapted and simplified from the scales used, and the questionnaire was standardized to a five-point Likert scale ranging from strongly disagree (1) to strongly agree (5). Parental emotion regulation, outdoor intention, and self-control scales were completed by the parents themselves. The Emotion Regulation Questionnaire (Gross and John, 2003) and Difficulties in Emotion Regulation Scale (Gratz and Roemer, 2004) were used for emotion regulation. The Parental Attitude Toward Nature Scale (Lene’McFarland, 2010) and Behavior and Attitudes Questionnaire for Healthy Habits (Henry et al., 2013) were used for outdoor intention. Self-control was measured using the Self-Control Scale (Tangney et al., 2004). Unlike adolescents, who possess the ability to self-reflect, children are unable to complete the test independently and are best measured based on caregiver reports (Domoff et al., 2019). Therefore, the parent-reported Child Behavior Checklist for Ages 1.5–5 was used to measure depression and social withdrawal in children (Ivanova et al., 2010). To measure their smart device addiction, we used the parent-reported Problematic Media Use Measure Scale (Domoff et al., 2019). Since the questionnaire was in English, two graduate students edited it to ensure the accuracy of the language. Participants were required to answer all the questions completely for the questionnaire to be submitted successfully. After the questionnaire was designed, we conducted a pilot survey with the parents of 50 pre-schoolers to ensure that the questions were reasonable; based on the results, we reworked the questionnaire. Supplementary Appendix A presents the final questionnaire questions.

We conducted our research in Linfen, China, because it values preschool education, having had a gross preschool enrollment rate of 98.5% in 2021 (Linfen Daily, 2022), which is well above the national average of 88.1% (People’s Daily, 2022). Questionnaires were distributed to parents of preschool children in Linfen from July 11 to July 18, 2022. Through instant messaging software, we accessed a local chat group of preschoolers’ parents. Parents usually share their parenting stories and experiences, sometimes even asking for help from other parents in the group. We randomly approached 50 parents to fill out the questionnaire and requested them to forward it to six more parents to fill out. The purpose of the study was communicated before handing out the questionnaires, and consent was obtained from parents under the condition of keeping their details anonymous. Parents received a CNY 10 shopping coupon each upon submission of the questionnaire. Three hundred questionnaires were distributed, out of which we received 281 responses. After removing the invalid responses, 236 responses were finally selected for analysis.

### 4.2. Data analyses methods

As this was an exploratory study with six variables and a relatively small sample size, we chose the partial least squares equation modeling (PLS-SEM) method for analysis, as it is suitable for small sample exploration, can measure more than six variables, and is convenient for handling non-normally distributed data (Hair et al., 2017).

Data distribution was measured by multivariate normality analysis using a web calculator^1^ (accessed on July 22, 2022). The following results were obtained: Mardia’s multivariate skewness (*β* = 258.567, *p* < 0.001) and multivariate kurtosis (*β* =1273.900, *p* < 0.001), which suggested multivariate non-normality.

The PLS-SEM analysis method has been widely used in the field of early childhood education (Yu and Wang, 2020; Kaur and Sharma, 2021; Kong and Yasmin, 2022). In particular, there are precedents in the literature for using this method to analyze children’s smart device use (Lee et al., 2022). Therefore, we analyzed the data in this study using PLS-SEM (Cao et al., 2021).

## 5. Results

### 5.1. Demographics

To better understand the population classifications, the following statistics were used in this study: A total of 236 (185 mothers and 51 fathers) completed questionnaires were collected.

The respondents were under 25 years old (*N* = 29, 12.3%), 25–30 years old (*N* = 56, 23.7%), 30–35 years old (*N* = 76, 32.2%), 35–40 years old (*N* = 41, 17.4%), and over 40 years old (*N* = 34, 14.4%). Of these, 32 (13.6%) had completed high school or below, 82 (34.4%) had a junior college degree, 76 (32.2%) had a bachelor’s degree, and 46 (19.5%) had a master’s degree or above. Children (125 boys and 111 girls) were 2 years old (*N* = 60, 25.4%), 3 years old (*N* = 48, 20.3%), 4 years old (*N* = 62, 26.3%), and 5 years old (*N* = 66, 28%).

### 5.2. Bias test results

The PLS analysis should be preceded by checking for non-response bias. Non-response bias usually occurs when some respondents are unable to participate accurately in the survey, resulting in an under-represented sample. Non-response groups can produce misleading findings that cannot be generalized to the entire target group and are thus under-represented (Berg, 2005). Therefore, the problem of non-response before, during, and after data collection must be considered (Van der Stede et al., 2005).

The reasons for non-response bias are as follows: first, the respondents may not have been capable of answering, for example, the respondent was ill or disabled; second, respondents were competent but deviated in filling out their answers due to lack of time or carelessness; third, the respondents were uncooperative and refused to take part in the survey; fourth, respondents were concentrated in one group, resulting in a lack of other types of representative samples in the survey.

To minimize non-responses, participants should be informed in advance and provided incentives before and during data collection (Rogelberg and Stanton, 2007). Therefore, the following measures were undertaken in this study. First, instructions were issued that all the questions in the questionnaire must be answered. Second, coupons were issued to the participants. Third, non-response bias can usually be measured with a *t*-test (Salehan et al., 2017); therefore, we performed paired T-tests on the demographic data of the initial as well as the final 25 individuals who completed the questionnaire and found no significant variance between the means of the two groups. Therefore, the non-response bias in this study is not a cause for concern (Rogelberg and Stanton, 2007; Salehan et al., 2017).

Second, we measured the common method bias (CMB) of the data using the methods of Podsakoff et al. (2003) and Kock (2015). The results indicated that the rate of extracting a single factor in Podsakoff et al.’s (2003) measure was 36.05%, which was below the threshold of 40%. In the PLS-SEM measurement method, the variance inflation factor (VIF) values were below the threshold of 3.3 (Sharma et al., 2021). This ensured that the common method deviation in this study satisfied the requirements.

### 5.3. Measurement model

In this study, composite reliability (CR), average variance extracted (AVE), discriminatory validity, and outer loading were used to ensure the quality of the model. The results showed that the CR and Cronbach’s alpha of the variables in the data were greater than 0.7, ensuring the internal consistency of the data. The AVEs of the variables in the data and the external loadings were all greater than 0.5 and 0.7, respectively, ensuring that the convergent validity of the data met the requirements (Hair et al., 2017), as shown in Table 1.

**Table 1:** Reliability and validity of constructs.

Latent variable	Item	Loading	Mean (SD)	Cronbach’s α	CR	AVE	*R* ^2^
PER	PER1	0.737	2.165 (0.662)	0.844	0.885	0.562	-
	PER2	0.751					
	PER3	0.727					
	PER4	0.733					
	PER5	0.776					
	PER6	0.771					
PSC	PSC1	0.737	2.117 (0.709)	0.85	0.893	0.625	0.433
	PSC2	0.741					
	PSC3	0.821					
	PSC4	0.819					
	PSC5	0.831					
POI	POI1	0.722	2.136 (0.728)	0.822	0.874	0.582	0.306
	POI2	0.771					
	POI3	0.751					
	POI4	0.791					
	POI5	0.777					
CDE	CDE1	0.733	3.586 (0.804)	0.84	0.887	0.611	0.219
	CDE2	0.796					
	CDE3	0.802					
	CDE4	0.804					
	CDE5	0.770					
CSW	CSW1	0.786	3.811 (0.760)	0.843	0.888	0.614	0.226
	CSW2	0.786					
	CSW3	0.779					
	CSW4	0.804					
	CSW5	0.761					
CSD	CSD1	0.822	3.532 (0.910)	0.906	0.928	0.681	0.397
	CSD2	0.826					
	CSD3	0.844					
	CSD4	0.826					
	CSD5	0.806					
	CSD6	0.827					

PER—Parental emotion regulation ability; PSC—Parental self-control ability; POI—Outdoor intention; CDE—Child depression; CSW—Child social withdrawal symptoms; CSD—Child smart device addiction.

To identify discriminant validity, we use the Fornell and Larcker and the heterotrait–monotrait ratio (HTMT) tests. The results indicated that the square root of each variable’s AVE was greater than its correlation with other variables (Hair et al., 2017), HTMTs were below 0.85, ensuring that the data discriminant validity was met (Hair et al., 2017), as shown in Table 2.

**Table 2:** Discriminant validity.

	PER	PSC	POI	CDE	CSW	CSD
Fornell–Larcker Criterion						
PER	0.749					
PSC	0.658	0.791				
POI	0.554	0.654	0.763			
CDE	−0.468	−0.497	−0.443	0.781		
CSW	−0.475	−0.533	−0.469	0.626	0.783	
CSD	−0.393	−0.403	−0.366	0.583	0.544	0.825
Heterotrait–Monotrait Ratio						
	PER	PSC	POI	CDE	CSW	CSD
PER						
PSC	0.774					
POI	0.657	0.774				
CDE	0.552	0.586	0.526			
CSW	0.561	0.633	0.551	0.736		
CSD	0.446	0.453	0.411	0.662	0.615	

PER—Parental emotion regulation ability; PSC—Parental self-control ability; POI—Outdoor intention; CDE—Child depression; CSW—Child social withdrawal symptoms; CSD—Child smart device addiction.

### 5.4. Structural model

We first tested for covariance, and the results showed that the VIFs in the variables were all less than 3 and met the requirements. Then, we used a structural model to test the hypotheses. *β*s > 0 meant a positive influence, and *β*s < 0 meant a negative influence. A result with a value of *p* < 0.05 was referred to as significant. The path coefficients and significance test results are shown in Table 3.

**Table 3:** Assessment of the structural model.

Hypothesis	*β*	STDEV	T-statistic	*p*-Value	Result
H1a: PSC - > CSD	−0.051	0.087	0.583	0.560	Reject
H1b: POI - > CSD	−0.061	0.087	0.702	0.482	Reject
H2a: CDE - > CSD	0.381	0.088	4.312	0.000	Support
H2b: CSW - > CSD	0.256	0.086	2.977	0.003	Support
H3a: PER - > PSC	0.658	0.05	13.058	0.000	Support
H3b: PER - > POI	0.554	0.05	11.123	0.000	Support
H4a: PER - > CDE	−0.468	0.062	7.551	0.000	Support
H4b: PER - > CSW	−0.475	0.059	8.046	0.000	Support
Edu - > F-CSD	−0.006	0.057	0.111	0.912	-
Parentalage - > F-CSD	−0.097	0.055	1.78	0.075	-
Childage - > F-CSD	−0.039	0.051	0.777	0.437	-
Childsex - > F-CSD	0.02	0.107	0.189	0.85	-
Parentalsex - > F-CSD	−0.217	0.115	1.892	0.059	-

The results show that parental self-control had no significant influence on children’s smart device addiction (*β* = −0.051, *p* = 0.560); therefore, H1a is not supported. The influence of parents’ outdoor intention on their children’s smart device addiction (*β* = −0.061, *p* = 0.482) was also not significant, due to which H1b is not supported. However, there was a significant positive influence of child depression (*β* = 0.381, *p* < 0.001) and social withdrawal (*β* = 0.256, *p* = 0.003) on children’s smart device addiction, supporting H2a and H2b, respectively.

Also, parental emotion regulation had a significant positive influence on parental self-control (*β* = 0.658, p < 0.001) and parental outdoor intention (*β* = 0.554, *p* < 0.001), supporting H3a and H3 b, respectively. Furthermore, parental emotion regulation had a significant negative influence on child depression (*β* = −0.468, *p* < 0.001) and children’s social withdrawal (*β* = −0.475, *p* < 0.001), supporting H4a and H4b, respectively.

To ensure the goodness of fit (GOF) of the model, we used standardized root mean square residuals (SRMRs). The results showed that the SRMR was less than 0.08, which meant that the fit met the requirements (Benitez et al., 2020).

### 5.5. Mediation effect

According to the protection-risk model proposed in this study, the variables may have a mediating effect on the relationship between parental emotion regulation and children’s smart device addiction. Therefore, an additional mediation analysis was necessary to examine the mediating effects of parental self-control and outdoor intention as well as children’s social withdrawal and depression between parental emotion regulation and children’s smart device addiction.

We analyzed the mediating effects in the model using SmartPls, as shown in Table 4, and a result with a value of *p* < 0.05 was referred to as significant. Children’s social withdrawal and depression mediate the effect of parental emotion regulation on their smart device addiction. However, neither parental self-control nor their outdoor intention mediates the effect of parental emotion regulation on young children’s smart device addiction.

**Table 4:** Mediation effect results.

Path	*ß*	STDEV	T Statistics	*p* Values
PER - > CSW - > CSD	−0.122	0.045	2.683	0.007
PER - > PSC - > CSD	−0.033	0.059	0.571	0.568
PER - > POI - > CSD	−0.034	0.05	0.683	0.495
PER - > CDE - > CSD	−0.178	0.054	3.336	0.001

## 6. Discussion and conclusion

### 6.1. Key findings

First, parental emotion regulation has a negative influence on children’s depression and social withdrawal (Han and Shaffer, 2012; Frigerio et al., 2022) because negative emotions affect parenting and responses to negative reactions. Parental emotion regulation influences the quality, timeliness, and frequency of family member interactions, and high levels of parental self-regulation can reduce social withdrawal and depression in children (Crandall et al., 2015; Gouveia et al., 2018). Conversely, parents’ negative emotions may lead them to be hypersensitive, avoidant, punitive, overly controlling, and focused on themselves rather than their children’s concerns, which may further disrupt cooperative interactions (Dix, 1991). Children’s reluctance to interact with their parents increases the likelihood of depression and social withdrawal.

Second, children’s depression and social withdrawal have a significant positive influence on children’s smart device addiction. Such findings are consistent with previous studies showing that the more severe the depressive symptoms, the more likely the addition to smart devices (Andreassen et al., 2016; Bui et al., 2021; Mun and Lee, 2021). Social phobia, isolation, and other issues can increase the likelihood of addiction to smartphones and the Internet (Estévez et al., 2017; Poulain et al., 2019; Serra et al., 2021; Diotaiuti et al., 2022). Smart devices allow individuals to escape from reality for a short time, soothe negative emotions, and provide psychological support (Serra et al., 2021). In particular, when there are psychological problems such as depression and social withdrawal, there is a greater tendency to use smart devices to relieve them, thus, exacerbating the duration of smart device use and thereby triggering the risk of addiction to it.

The results of this study demonstrate that emotional regulation ability positively affects parents’ outdoor intention. Previous research has identified that positive emotions promote intrinsic outdoor motivation (Løvoll et al., 2017). People with high levels of emotion regulation are more likely to maintain positive emotions, thereby promoting outdoor intentions. However, this study did not confirm that parental outdoor intention reduces smart device addiction in preschoolers, which could be due to parents having a certain fear of outdoor play being harmful and believing that their children are safer and more secure at home (Peck, 2012). In particular, as China is still in the prevention and control stage of coronavirus disease 2019 (COVID-19), preschool children have low resistance, and therefore, parents are worried that outdoor activities will be detrimental to their children’s health. Therefore, despite a high parental intention to be outdoors, the actual time allowed for preschoolers to be outdoors is still limited, so children still spend most of their time at home, thus facing the potential risk of becoming addicted to smart devices.

The present study also confirmed the positive influence of emotion regulation on parental self-control, which is consistent with previous findings. There is a correlation between emotions and self-control (Tice and Bratslavsky, 2000; Chester et al., 2016); that is, the better an individual’s ability to regulate emotions, the higher their self-control. However, the influence of parental self-control on reducing children’s smart device addiction was not confirmed in this study, which may be because the parental role-modeling factor is only one of the protective influences (Jessor et al., 2003), and children’s level of self-control is also influenced by several other factors such as parenting, biological, and social structural factors (Wright and Beaver, 2005; Beaver et al., 2007; Buker, 2011). Addiction is also influenced by the dopamine system and genetic factors (Febo et al., 2017; Blum et al., 2022). Therefore, although the modeling effect of parental self-control influences that of children, the inhibitory effect on children’s smart device addiction was weaker than expected.

This study found that children’s depression and social withdrawal mediated the relationship between parental emotion regulation and children’s smart device addiction. Parental emotional regulation can reduce children’s smart device addiction by reducing depression and social withdrawal. Therefore, this study confirms the importance of emotion regulation in parenting and children’s psychological health (Crandall et al., 2015; Gouveia et al., 2018; Frigerio et al., 2022).

### 6.2. Theoretical contributions

First, although parental influence on children’s smart device addiction has been addressed in prior studies, research has mostly been conducted from the perspective of parenting and parents’ own smart device use (McDaniel and Radesky, 2020; Lee et al., 2022; Lee and Kim, 2022; Yang et al., 2022). It has neither been verified by other factors, such as emotional regulation, self-control, and outdoor intention, nor has it considered child psychological factors, such as depression and social withdrawal. Second, this study applied the protection–risk model designed for studying adolescents’ smart device addictive behavior in preschoolers, determining the influence of risk factors (depression, social withdrawal) on children’s smart device addiction. This confirmed that the protection–risk model can also be applied to the analysis of addictive behavior on smart devices in preschoolers, thus, expanding the use of the theory and enriching its connotation.

By introducing parental emotion regulation variables into the protection–risk model, this study confirms that parental emotion regulation can have an impact on model protective factors (self-control, outdoor intention) and vulnerable risk factors (depression, social withdrawal). Such results enrich the antecedents of the protection–risk model and contribute to the development of the theory.

### 6.3. Practical contributions

This study also provides practical recommendations for parents to prevent and reduce their children’s addiction to smart devices.

First, parents can set a time for their children to use smart devices. They can refer to the recommendations of the WHO to shorten the length of time that children use smart devices each day and minimize their solo use by them (Bozzola et al., 2018; WHO, 2019). Parents using devices together with their children can better control their children’s usage time and help them filter out content that is not beneficial for them, enabling them to use their smart devices wisely.

Second, parental emotion regulation plays an important role in children’s social withdrawal and depression (Han and Shaffer, 2012; Crespo et al., 2017). Parents can try to regulate their own negative emotions in a timely manner to avoid the same in their children, which increases social withdrawal and depression in them. When children experience social withdrawal and depression, parents can try to communicate with them to understand the cause and provide guidance to alleviate it. This will prevent children from becoming addicted to their smart devices due to social withdrawal and depression.

Third, parents need to balance the distribution of indoor and outdoor as well as solo and social activities for their children (Adebar, 2018). Parents can participate in outdoor activities with their children (Adebar, 2018), which will enrich their children’s daily lives while possibly reducing the amount of time they spend using smart devices, thereby avoiding their addiction to them.

### 6.4. Limitations and future directions

There are some limitations in this study. First, this study did not consider a large enough sample size, and therefore, the representativeness of the results may be problematic. Therefore, in the future, a larger sample needs to be considered. Second, the findings may differ from those of western countries owing to cultural and educational differences. In the future, it will be necessary to include samples from other cities in China, as well as other countries, and compare the results of the study. Third, as a quantitative study, this could not provide detailed insight into parents’ thoughts; therefore, a qualitative analysis can be attempted to gain more insight into the influence of parental emotion regulation on children’s smart device addiction. Fourth, this study only assessed parents and did not measure the influence of neighbors, kindergarten teachers, or peers on preschoolers’ smart device addiction. Future analysis of various other factors influencing smart device addiction in preschool children is encouraged. Finally, because of the COVID-19 pandemic, people’s outdoor activities have been limited, which may have biased the investigation in this study, and we hope that further studies will be conducted after the pandemic.

## Data availability statement

The raw data supporting the conclusions of this article will be made available by the authors, without undue reservation.

## Ethics statement

Ethical review and approval was not required for the study on human participants in accordance with the local legislation and institutional requirements. Written informed consent for participation was not required for this study in accordance with the national legislation and the institutional requirements.

## Author contributions

LC and JC: conceptualization and validation. LC: methodology, software, investigation, resources, data curation, writing—original draft preparation, and visualization. JC: formal analysis, writing—review and editing, supervision, and project administration. All authors have read and agreed to the published version of the manuscript.

## Conflict of interest

The authors declare that the research was conducted in the absence of any commercial or financial relationships that could be construed as a potential conflict of interest.

## Publisher’s note

All claims expressed in this article are solely those of the authors and do not necessarily represent those of their affiliated organizations, or those of the publisher, the editors and the reviewers. Any product that may be evaluated in this article, or claim that may be made by its manufacturer, is not guaranteed or endorsed by the publisher.

## Footnotes

^1^ https://webpower.psychstat.org/
