# Supplementary material for: Factors influencing smart device addiction among preschool children: An extended protection-risk model perspective
Source: Front Psychol. 2023 Feb 9;14:1017772. doi: 10.3389/fpsyg.2023.1017772 (PMC9947858; doi:10.3389/fpsyg.2023.1017772)
Supplement: Supplementary file 1 [file Table_1.docx]

| Factor | Serial Num | Item | Reference |
| --- | --- | --- | --- |
| Parental emotion regulation  （PER) | PER1 | When I want to feel fewer negative emotions (such as sadness or anger), I change what I’m thinking about. | Gross & John (2003)  and Gratz & Roemer (2004) |
|  | PER2 | When I’m faced with a stressful situation, I make myself think about it in a way that helps me stay calm. |  |
|  | PER3 | I am clear about my feelings. |  |
|  | PER4 | When I’m upset, I can still get things done. |  |
|  | PRE5 | When I’m upset, I feel like I can remain in control of my behavior. |  |
|  | PER6 | When I’m upset, I know that I can find a way to eventually feel better. |  |
| Parental self-control (PSC) | PSC1 | I can finish the work on time. | Tangney et al. (2004) |
|  | PSC2 | I can stop myself from doing something if I know it is wrong. |  |
|  | PSC3 | People would say that I have iron self-discipline. |  |
|  | PSC4 | Pleasure and fun can’t keep me from getting work done. |  |
|  | PSC5 | I am able to work effectively toward long-term goals. |  |
| Parental outdoor intention（POI) | POI1 | People should spend more time outside. | Lene'McFarland (2010) and Henry et al. (2013) |
|  | POI2 | Spending time outdoors is an enjoyable alternative to smart devices. |  |
|  | POI3 | I enjoy eating meals outdoors. |  |
|  | POI4 | Family vacations are a good opportunity to spend time outdoors. |  |
|  | POI5 | I usually join my child in outdoor and play activities. |  |
| Child depression  (CDE) | CDE1 | My child clings to adults or is too dependent. | Ivanova et al. (2010) |
|  | CDE2 | My child’s feelings are easily hurt. |  |
|  | CDE3 | My child is self-conscious or easily embarrassed. |  |
|  | CDE4 | My child looks unhappy without good reason. |  |
|  | CDE5 | My child is unhappy, sad, depressed. |  |
| Child social withdrawal  (CSW) | CSW1 | My child avoids looking others in the eye |  |
|  | CSW2 | My child doesn’t answer when people talk to him/her. |  |
|  | CSW3 | My child shows little interest in things around him/her. |  |
|  | CSW4 | My child is withdrawn, doesn’t get involved with others. |  |
|  | CSW5 | My child seems unresponsive to affection. |  |
| Child smart device addiction（CSD) | CSD1 | It is hard for my child to stop using their smart device. | Domoff et al. (2019) |
|  | CSD2 | My child becomes frustrated when he/she cannot use their smart device. |  |
|  | CSD3 | The amount of time my child wants to use a smart device keeps increasing. |  |
|  | CSD4 | The ﬁrst thing my child asks to do when he/she comes home from school is to use their smart device. |  |
|  | CSD5 | When my child has had a bad day, the smart device seems to be the only thing that helps him/her feel better. |  |
|  | CSD6 | My child’s smart device use interferes with family activities. |  |
